# Predicting ambulatory energy expenditure in lower limb amputees using multi-sensor methods

**DOI:** 10.1371/journal.pone.0209249

**Published:** 2019-01-31

**Authors:** Peter Ladlow, Tom E. Nightingale, M. Polly McGuigan, Alexander N. Bennett, Rhodri D. Phillip, James L. J. Bilzon

**Affiliations:** 1 Department for Health, University of Bath, Bath, United Kingdom; 2 Academic Department of Military Rehabilitation, Defence Medical Rehabilitation Centre (DMRC) Headley Court, Surrey, United Kingdom; 3 National Heart and Lung Institute, Faculty of Medicine, Imperial College London, London, United Kingdom; University of Illinois at Urbana-Champaign, UNITED STATES

## Abstract

**Purpose:**

To assess the validity of a derived algorithm, combining tri-axial accelerometry and heart rate (HR) data, compared to a research-grade multi-sensor physical activity device, for the estimation of ambulatory physical activity energy expenditure (PAEE) in individuals with traumatic lower-limb amputation.

**Methods:**

Twenty-eight participants [unilateral (n = 9), bilateral (n = 10) with lower-limb amputations, and non-injured controls (n = 9)] completed eight activities; rest, ambulating at 5 progressive treadmill velocities (0.48, 0.67, 0.89, 1.12, 1.34m.s^-1^) and 2 gradients (3 and 5%) at 0.89m.s^-1^. During each task, expired gases were collected for the determination of $\dot{V}O_{2}$ and subsequent calculation of PAEE. An Actigraph GT3X+ accelerometer was worn on the hip of the shortest residual limb and, a HR monitor and an Actiheart (AHR) device were worn on the chest. Multiple linear regressions were employed to derive population-specific PAEE estimated algorithms using Actigraph GT3X+ outputs and HR signals (GT3X+HR). Mean bias±95% Limits of Agreement (LoA) and error statistics were calculated between criterion PAEE (indirect calorimetry) and PAEE predicted using GT3X+HR and AHR.

**Results:**

Both measurement approaches used to predict PAEE were significantly related (*P*<0.01) with criterion PAEE. GT3X+HR revealed the strongest association, smallest LoA and least error. Predicted PAEE (GT3X+HR; unilateral; *r* = 0.92, bilateral; *r* = 0.93, and control; *r* = 0.91, and AHR; unilateral; *r* = 0.86, bilateral; *r* = 0.81, and control; *r* = 0.67). Mean±SD percent error across all activities were 18±14%, 15±12% and 15±14% for the GT3X+HR and 45±20%, 39±23% and 34±28% in the AHR model, for unilateral, bilateral and control groups, respectively.

**Conclusions:**

Statistically derived algorithms (GT3X+HR) provide a more valid estimate of PAEE in individuals with traumatic lower-limb amputation, compared to a proprietary group calibration algorithm (AHR). Outputs from AHR displayed considerable random error when tested in a laboratory setting in individuals with lower-limb amputation.

## Introduction

Individuals who experience traumatic lower-limb amputation have been shown to be at increased risk of developing cardiometabolic diseases [1–3]. Reducing the development of unfavourable body composition changes [4–6] and the secondary health conditions associated with obesity, such as cardiovascular disease and type 2 diabetes mellitus [1–3], is a primary objective in the long-term recovery of individuals with lower-limb amputation. It is well established that the higher the level of amputation (above knee versus below knee) and the greater number of lower-limbs amputated (bilateral versus unilateral) are associated with a higher metabolic cost of walking and reduced ambulatory physical activity (PA) [7–9]. However, little is known about the consequences of amputation severity on habitual PA levels or ambulatory physical activity energy expenditure (PAEE). Consequently, PA requirements for the maintenance or improvement of metabolic health and protection against chronic degenerative diseases, is poorly understood in this population. Severe lower-limb injuries have also been associated with high levels of mental health disorders, such as moderate to severe depression (38%) and anxiety (29%) [10]. Strategies to mitigate the risk or managing these conditions are of upmost importance. Exercise and PA interventions aimed at improving function, health and wellbeing in individuals with amputation may benefit from the use of objective PA measurements.

Criterion or ‘gold standard’ measures of energy expenditure (i.e. indirect calorimetry and doubly labelled water) are highly accurate, but relatively expensive, requiring sophisticated equipment rendering them impractical to use outside of the laboratory when assessing free-living PA. The ability to detect subtle or large variances in the duration spent at different PA levels during free-living conditions, patterns of PAEE (morning, afternoon, evening) and exercise intensity are not possible using doubly labelled water technique (only total energy expenditure) during a monitoring period. Pedisic and Bauman [11] suggested that accelerometer-assessed PAEE, using algorithms intrinsic to certain devices may not be generalizable to a target population, an important consideration for individuals predisposed to significant gait deficiencies such as individuals with lower-limb amputation [12, 13]. Therefore, the logical first step prior to using objective devices in surveillance research is to ensure that the method has been validated in the population of interest.

Research in the area of assessing PA in individuals with amputation has previously relied on subjective amputation specific self-reported questionnaires [14, 15] in conjunction with objective measures of functional mobility (e.g. step count and timed up and go). Limitations to using self-reported measures of PA are well-known and include inaccurate subjective reporting and recall bias [16]. A systematic review [17] of instruments used for the assessment of PA in individuals with amputation demonstrated that most instruments are not specific enough for the population (i.e. they do not account for the loss of articular structures and sensory/motor function in the lower-extremity) [18]. Therefore, previous instruments have been unable to discriminate for the known differences in PAEE between persons with and without lower-limb amputation, thereby potentially underestimating the ‘actual’ PA levels of this population. Having access to an objective measurement of PAEE can facilitate our understanding of a number of other clinically important areas with greater accuracy than we might have had before. Recently, our group demonstrated that the Actigraph GT3X+ accelerometer, worn at the hip on the shortest residual limb, provides a valid estimation of PAEE in individuals with unilateral and bilateral traumatic lower-limb amputation [19]. However, accelerometry data alone doesn’t capture the physiological strain associated with certain ambulatory behaviours, such as walking up a gradient [20]. While this initial exploration into the objective measurement of PAEE in individuals with amputation is positive, multi-sensor devices, incorporating physiological signals, might offer a greater improvement in prediction accuracy [21]. Heart rate is known to be a valuable physiological signal in the estimation of energy expenditure due to its near linear relationship, also the relative success of using the Physiological Cost Index (PCI) in quantifying energy expenditure among individuals with lower limb amputation [22, 23].

Wearable PA monitors (e.g. Fitbit, Microsoft Band and Apple Watch) are growing in popularity and provide an opportunity for large numbers of the public to self-monitor their own PA behaviours [24]. Albert *et al*. [25] performed a feasibility study monitoring daily function in persons with trans-femoral amputations using a commercial activity monitor (Fitbit). They found that this monitor has the potential to be used to assess the PA levels of people with lower-limb amputation. However, commercially available activity monitors have not been specifically validated in individuals with amputation, which is a concern for using such multi-sensor devices in this population.

The Actiheart (AHR) is a research-grade multi-sensor device which incorporates heart rate (HR) and accelerometry measurements to predict PAEE [26]. It has been widely used to measure free-living PA in able-bodied individuals but further research in diverse populations, such as individuals with amputation, is warranted. It is unknown whether the proprietary predictive algorithms of the AHR can accurately predict ambulatory PAEE in persons who have experienced amputation. It is also unknown whether the combination of Actigraph GT3X+ accelerometer and HR data (GT3X+HR) could be used to derive a predictive algorithm with comparable or superior accuracy for the estimation of PAEE. This study aims to test the hypothesis that a bespoke algorithm (GT3X+HR) would demonstrate greater validity and lower random error in predicting PAEE in persons with lower-limb amputations, compared with the proprietary predictive algorithms of the AHR device.

## Materials and methods

### Research ethics approval

Ethical approval was granted by the United Kingdom Ministry of Defence Research Ethics Committee (MODREC) and written informed consent was obtained from each participant.

### Sample size

An *a priori* power calculation was performed based on data from a previous study in spinal cord injured humans [27]. It was estimated that a minimum of 8 participants would be required to detect a statistically significant difference in mean absolute error between the Actiheart^TM^ propietry predictive algorithms (mean absolute error 51.4±38.9%) and a bespoke individually calibrated algorithm (mean absolute error 16.8±15.8%), giving an estimated effect size of (Cohen *d*) of 1.0. The power was set at 0.8 and the alpha at 0.05. Given the distinct challenges in recruiting and retaining participants from unique clinical populations, we anticipated a ~20% drop-out rate and aimed to recruit 10 participants per group to achieve a final sample of at least 8.

### Participants

A final sample of UK military personnel with traumatic unilateral (n = 9) and bilateral (n = 10) lower-limb amputation/s and nine non-injured healthy male controls volunteered to participate in this study. All participants were male and visited the Military Performance and Rehabilitation Laboratory (MPARL) at the Defence Medical Rehabilitation Centre (DMRC), Headley Court, one morning after a 10 hour overnight fast (including abstinence from caffeine and physical activity). Inclusion criteria included all injured participants having experienced traumatic amputation and had previously received at least three 4-week admissions of intensive exercise rehabilitation at DMRC Headley Court from an interdisciplinary team of health professionals [28]. All patients received a prosthetic fitting prior to commencing the trial and had been given clearance to ambulate on a treadmill by their physiotherapist. Exclusion criteria were based upon the participant's medical history (screened by their physician). This includes severe traumatic brain injury, medication that alters heart rate variability, and any mobility restricting conditions, such as painful heterotopic ossification or insufficient wound healing around the stump. The control group are non-injured physically active men (civilian and military who engage in aerobic or resistance based training at least three times per week). The clinical population being tested are all UK military personnel with traumatic lower-limb amputation/s who (pre-injury) would have been active non-injured adults. By using an age-matched active male adult population as a control we are able to comment on the impact of losing a limb and the ability of proprietary algorithms ability to detect such a change against normative activity data from their non-injured peers.

### Indirect calorimetry

Participants wore a sealed face mask and expired gases were analysed using a portable metabolic system (Metamax 3B, Cortex, Leipzig, Germany), which has good accuracy compared to other portable metabolic systems [29]. Expired gases passed through a flow meter and are channelled down a sampling line into the analyser unit where the fractions of O_2_ and CO_2_ in expired gases are measured. Metabolic data were retrieved and analysed using the Metamax software. $\dot{V}O_{2}$ steady state can be achieved within 3 minutes [30]. Oxygen uptake ($\dot{V}O_{2}$) and carbon dioxide production (CO_2_) were used to estimate energy expenditure (kcal·min^-1^) during the final two minute of each five minute activity. The Metamax was calibrated according to manufacturer’s instructions prior to use.

#### Activity monitors

Participants wore an AHR monitor (Actiheart, Cambridge Neurotechnology Ltd, UK), which integrates accelerometer and HR signals to derive PAEE. The unit was fitted using two adhesive ECG chest electrodes, according to the manufacturer’s instructions. The AHR unit has been described previously [26]. AHR devices were initialised to long-term recording with 30 second epochs at a sampling frequency of 32 Hz. PAEE was calculated using Branched Model equations [31].

An Actigraph GT3X+ tri-axial accelerometer (Actigraph, Pensacola, FL, USA) was worn around the waist, above the hip (along the anterior axillary line) on the side of the shortest residual limb, as recommended by Ladlow *et al*. [19], using an elasticated belt. Following the Nyquist principle, the devices were initialised with a sampling frequency of 30 Hz, thereby allowing the capture of general human movement [32] and a similar sampling frequency to the AHR monitor. The componentry and capabilities of the Actigraph GT3X+ are described elsewhere. [33] A Polar T31 HR monitor (Polar Electro Inc., Lake Success, NY, USA) was firmly secured on the chest using an elastic strap and ultrasound gel was applied to the electrodes to improve the connection. HR transmitted by the Polar T31 was captured by a wireless receiver module connected to the Metamax 3B.

#### Testing protocol

The testing protocol is described in more detail elsewhere [19]. The Metamax 3B and the two activity monitors were synchronised before use and a Polar HR monitor was worn throughout the protocol. Resting metabolic rate (RMR; kcal·day^-1^) was measured between 08–00 to 09:00 in a semi-recumbent position in accordance with best practice guidelines [34]. Following the measurement of RMR and anthropometric assessment (i.e. body stature, body mass, waist and hip circumference), participants completed a walking protocol on a treadmill (Woodway Desmo, USA). This protocol consisted of ambulating at 5 progressive velocities 0.48, 0.67, 0.89, 1.12, 1.34 m.s^-1^ (1, 1.5, 2, 2.5 and 3 mph) and 2 gradients (3% and 5%) at 0.89 m.s^-1^ (2 mph). Each activity lasted 5-minutes with no recovery between each velocity. Participants were asked to complete the entire protocol without resting their arms on the handrail. Participants were told to stop if they experience residuum pain, prosthetic discomfort or difficulty maintaining the speed of the treadmill belt to a point where they felt they were at risk of falling.

PCI was calculated as the quotient of difference in working and resting heart rates and walking speed respectively self-selected (comfort) walking speed. The PCI value reflects the increased heart rate required for walking and is expressed as heart rate per metre by formula [22]:

*PCI = [mean HR at work – mean HR at rest] / walking velocity (m*.*min^-1^)*

Using the mean HR data from the final 2 minutes of each activity, we have modified this PCI equation using pre-determined velocities on the treadmill as oppose to comfortable self-selected walking speed as recommended by MacGregor [35].

#### Statistical analyses

Expired gas data were exported into Microsoft Excel from the Metamax 3B software. PAEE was then calculated using the $\dot{V}O_{2}$ and CO_2_ values (L·min^-1^), averaged over the final 2-min of each activity using the Weir equation [36]. As participants were fasted, dietary-induced thermogenesis was considered negligible and criterion PAEE was calculated by subtracting RMR (kcal·min^-1^) from total energy expenditure. Predicted PAEE was derived for both methods: GT3X+HR, by combining tri-axial accelerometer counts from the GT3X+ with HR using regression methods and; AHR, from the Actiheart software. Data from both derived methods were compared against criterion PAEE during the final two minutes of each activity (representative of steady-state).

AHR data was ascertained via entering the measured RMR (via indirect calorimetry), age, weight, height and sleeping HR (measured the night before testing) into the Actiheart software (Version 4.0.23), according to the manufacturer’s instructions. The GT3X+ accelerometer unit was downloaded using the ActiLife software (ActiGraph, Pensocola, FL, USA). PAC (counts·min^-1^) from the GT3X+, Polar HR (bpm) and AHR readings (kcal·min^-1^) were then averaged over the final two minutes of each activity. PAEE estimation models for the GT3X+HR were developed using corresponding data from each task, using multiple linear regression analyses. The dependent variable was indirect calorimetry PAEE (kcal·min^-1^). The independent variables were PAC (counts·min^-1^) from the GT3X+ with HR (bpm). Pearson product moment correlation coefficients (*r*) and coefficients of determination (R^2^) statistics were conducted to assess the association between the criterion PAEE and predicted PAEE for GT3X+HR, HR and AHR (AHR data; using proprietary group calibration). Standard Error of the Estimate (SEE) statistics was also calculated for each relationship. Ideally the population specific equation (GT3X+HR) would have been cross-validated using an independent sample. However, this is not always possible in hard to recruit populations, such as individuals with lower-limb amputations. Therefore, we adopted a leave-one-out ‘bootstrapping’ analysis [37], as performed and reported previously by Nightingale *et al*. (2015) [38].

Error statistics involved calculating the mean absolute error, mean absolute percentage error and mean signed error for each activity (displayed graphically using modified box and whisker plots), Bland-Altman plots with 95% limits of agreement analysis and root mean squared error (RMSE) displayed S1 Table. One-way ANOVA tests by group were performed with post-hoc Bonferroni corrections applied when comparing across 8 activities (rest, five progressive treadmill speeds and 2 gradients). Statistical significance was set a priori of *P*<0.05. All analyses were performed using IBM SPSS Statistics 21 for Windows (IBM, Armonk, NY, USA).

## Results

Demographic and anthropometric characteristics of the participants are described in Table 1. Criterion PAEE (kcal·min^-1^), GT3X+ accelerometer outputs, HR, AHR, and METs are displayed in Table 2. Mean criterion PAEE, GT3X+ PAC, HR, AHR PAEE, RPE and METs all increased with increasing treadmill velocity in the unilateral and control groups. In the bilateral group, between six and eight participants were unable to complete activities at the higher treadmill velocities and gradients, three individuals in the unilateral group were unable to complete the highest ambulatory velocity (1.34 m.s^-1^) which influenced the mean criterion values. There was a significant main effect in criterion PAEE, GT3X+ predicted PAEE, Actiheart, HR, METs and PCI in all groups (See Table 2 and S2 Table). In both amputation groups and control participants, the GT3X+HR model demonstrated the strongest relationships, smallest limits of agreement (LoA) (Table 3), mean absolute percentage errors and RMSE, compared to AHR (S1 Table).

**Table 1:** Demographic and anthropometric characteristics of the participants.

Variable	Unilateral		Bilateral		Control	
	Mean ± SD	Range	Mean ± SD	Range	Mean ± SD	Range
Number of Participants	9		10		9	
Age (years)	32 ± 5	23–41	29 ± 4	22–34	31 ± 6	25–45
Body Mass—without prosthesis (kg)	81 ± 11	63–108	82 ± 19	59–126	80 ± 7	68–89
Waist Circumference (cm) *	92 ± 13	75–115	100 ± 20	77–149	83 ± 4	76–90
Waist-hip ratio	0.90 ± 0.07	0.83–1.00	0.94 ± 0.09	0.86–1.17	0.86 ± 0.04	0.79–0.92
RMR (kcal·d^-1^)	1776 ± 269	1480–2158	1596 ± 178	1382–2051	1846 ± 191	1505–2059
Sleeping Heart Rate #	63 ± 9	46–77	57 ± 7	48–68	48 ± 3	45–55
Time Since Amputation (months) ǂ	23 ± 15	4–46	39 ± 14	21–61	-	
*Level of Amputation*:						
Below Knee	5		1		-	
Through Knee	2		2		-	
Above knee	2		3		-	
Bilateral: Below Knee and Above Knee	-		4		-	

*Significant difference between individuals with bilateral amputation and control group (*P*<0.05)

ǂ Significant difference between individuals with unilateral and bilateral amputation (*P*<0.05)

# Significant difference between control group and both amputation groups (*P*<0.05)

**Table 2:** Measured PAEE, accelerometer outputs at each anatomical location, calculated METs, RPE and number of participants for each activity (mean ± SD).

Activity	PAEE Metamax 3B (kcal·min^-1^)	GT3x+ (PAC·min^-1^)	HR	Actiheart (kcal·min^-1^)	METS (calculated)	RPE	RER	PCI	n
**Unilateral Amputation:** ǂ # ¶									
**RMR**	0.00 ± 0.00	0 ± 0	66 ± 11	0.00 ± 0.00	1.0 ± 0.0	6 ± 0	0.74 ± 0.04	-	9
**Treadmill 0.48 m.s**^**-1**^	2.40 ± 0.71	2643 ± 866	96 ± 17	1.03 ± 0.52	3.1 ± 0.8	8 ± 1	0.76 ± 0.04	1.01 ± 0.28	9
**Treadmill 0.67 m.s**^**-1**^	3.00 ± 0.95	2939 ± 908	101 ± 18	1.61 ± 0.74	3.6 ± 1.0	9 ± 1	0.79 ± 0.04	0.85 ± 0.24	9
**Treadmill 0.89 m.s**^**-1**^	3.61 ± 1.12	3353 ± 892	106 ± 21	2.15 ± 1.05	4.1 ± 1.1	11 ± 2	0.81 ± 0.03	0.74 ± 0.26	9
**Treadmill 1.12 m.s**^**-1**^	4.39 ± 1.42	4107 ± 707	112 ± 24	2.84 ± 1.93	4.7 ± 1.3	12 ± 2	0.83 ± 0.03	0.67 ± 0.26	9
**Treadmill 1.34 m.s**^**-1**^	5.89 ± 1.70	4977 ± 581	126 ± 36	4.01 ± 3.32	5.7 ± 1.8	12 ± 2	0.86 ± 0.05	0.73 ± 0.33	6
**Treadmill 3% (0.89 m.s**^**-1**^**)**	4.17 ± 1.06	3642 ± 981	111 ± 21	2.32 ± 1.15	4.5 ± 1.1	11 ± 1	0.82 ± 0.04	0.84 ± 0.26	9
**Treadmill 5% (0.89 m.s**^**-1**^**)**	4.82 ± 1.24	4020 ± 1005	119 ± 23	2.94 ± 2.09	5.0 ± 1.2	12 ± 2	0.84 ± 0.04	0.99 ± 0.29	9
Bilateral Amputation:* § ¥									
**RMR**	0.00 ± 0.00	0 ± 0	67 ± 10	0.00 ± 0.00	1.0 ± 0.0	6 ± 0	0.75 ± 0.04	-	10
**Treadmill 0.48 m.s**^**-1**^	3.72 ± 1.37	4800 ± 1410	108 ± 13	2.41 ± 1.40	4.4 ± 1.2	10 ± 2	0.78 ± 0.03	1.45 ± 0.32	10
**Treadmill 0.67 m.s**^**-1**^	4.59 ± 1.54	5264 ± 1603	121 ± 15	3.38 ± 1.96	5.1 ± 1.4	12 ± 2	0.81 ± 0.04	1.35 ± 0.29	10
**Treadmill 0.89 m.s**^**-1**^	5.46 ± 1.74	5600 ± 1502	133 ± 18	4.86 ± 2.81	5.8 ± 1.6	14 ± 3	0.84 ± 0.05	1.25 ± 0.32	10
**Treadmill 1.12 m.s**^**-1**^	5.54 ± 2.85	6123 ± 2823	139 ± 31	7.80 ± 5.00	5.3 ± 1.4	15 ± 3	0.91 ± 0.18	1.22 ± 0.43	3
**Treadmill 1.34 m.s**^**-1**^	5.23 ± 2.76	5235 ± 1212	142 ± 41	7.31 ± 4.14	5.7 ± 1.7	15 ± 0	0.86 ± 0.7	1.03 ± 0.36	2
**Treadmill 3% (0.89 m.s**^**-1**^**)**	5.93 ± 2.29	5973 ± 1592	138 ± 19	5.95 ± 4.25	6.1 ± 2	14 ± 3	0.87 ± 0.7	1.36 ± 0.40	8
**Treadmill 5% (0.89 m.s**^**-1**^**)**	5.77 ± 1.85	5806 ± 2231	146 ± 30	7.80 ± 5.26	5.7 ± 1.1	16 ± 2	0.93 ± 0.17	1.37 ± 0.56	4
**Control:**									
**RMR**	0.00 ± 0.00	0 ± 0	54 ± 4	0.00 ± 0.00	1.0 ± 0.0	6 ± 0	0.75 ± 0.04	-	9
**Treadmill 0.48 m.s**^**-1**^	1.43 ± 0.31	1299 ± 411	72 ± 7	1.34 ± 0.54	2.2 ± 0.3	7 ± 0	0.78 ± 0.03	0.61 ± 0.15	9
**Treadmill 0.67 m.s**^**-1**^	1.81 ± 0.32	1811 ± 368	74 ± 6	1.88 ± 0.65	2.5 ± 0.3	8 ± 1	0.82 ± 0.04	0.50 ± 0.09	9
**Treadmill 0.89 m.s**^**-1**^	2.32 ± 0.40	2430 ± 459	78 ± 4	2.22 ± 0.84	2.9 ± 0.4	9 ± 1	0.82 ± 0.05	0.45 ± 0.05	9
**Treadmill 1.12 m.s**^**-1**^	2.80 ± 0.43	3325 ± 480	82 ± 5	2.55 ± 0.84	3.3 ± 0.5	9 ± 1	0.82 ± 0.03	0.41 ± 0.04	9
**Treadmill 1.34 m.s**^**-1**^	3.40 ± 0.34	4144 ± 457	85 ± 5	2.91 ± 0.88	3.7 ± 0.4	10 ± 1	0.83 ± 0.04	0.38 ± 0.04	9
**Treadmill 3% (0.89 m.s**^**-1**^**)**	2.88 ± 0.38	2551 ± 385	83 ± 5	2.38 ± 0.92	3.3 ± 0.3	10 ± 1	0.84 ± 0.04	0.54 ± 0.06	9
**Treadmill 5% (0.89 m.s**^**-1**^**)**	3.45 ± 0.43	2720 ± 311	87 ± 4	2.52 ± 0.99	3.8 ± 0.3	10 ± 1	0.83 ± 0.04	0.61 ± 0.06	9

Not all participants with amputation/s were able to complete all of the treadmill speeds in this trial. The number of participant completers at each treadmill task is presented here. PAC from the GT3X+ combined with HR, HR alone and Actiheart data were all significantly (*P*<0.01) associated with criterion PAEE.

*Due to reduced participant numbers, all statistical analyses comparing the bilateral group with other groups were performed at speeds 0.48–0.89 m.s^-1^ and at 3% gradient.

ǂ A significant difference in criterion PAEE, HR and METs were only reported at higher intensities (1.12 m.s^-1^, 1.34 m.s^-1^ and 5% gradient at 0.89 m.s^-1^) between individuals with unilateral amputation and control group (*P*<0.05).

§ A significant differences in criterion PAEE, HR, METs, PAC (GT3X+ worn at the longest and shortest limb) and PCI were found between bilateral amputation versus the unilateral and control groups at all speeds analysed (*P*<0.05).

# Significant differences in PAC (GT3X+) were only reported at the lowest intensity of **0.48 m.s**^**-1**^ and the highest intensity of **1.34 m.s**^**-1**^ between individuals with unilateral amputation and control group (P<0.05).

¥ Significant differences in Actiheart outcomes were reported between the bilateral amputation and the unilateral amputation groups at all speeds analysed and at 0.89 m.s^-1^ and 3% gradient at 0.89 m.s^-1^ versus the control group.

¶ Significant differences in PCI were only reported at the lowest intensities (0.48 m.s^-1^ and 0.67 m.s^-1^) and highest intensities (1.34 m.s^-1^ and 0.89 m.s^-1^) between individuals with unilateral amputation and the control group

**Table 3:** The relationships between the three devices of the Actigraph GT3X+ with HR, HR alone Actiheart against criterion PAEE in all groups. Limits of agreement (LoA) expressed as mean ± 95% SD.

Location	*r*	R^2^	SEE (kcal·min^-1^)	LoA (kcal·min^-1^)	*P* Value
**Treadmill Walking**					
**Unilateral Amputation Group**					
GT3X+ and HR	0.92	0.85	0.78	0 ± 1.50	< 0.001
HR	0.89	0.79	0.91	0 ± 1.77	< 0.001
Acti-Heart	0.86	0.73	1.02	-1.40 ± 2.00	< 0.001
**Bilateral Amputation Group**					
GT3X+ and HR	0.93	0.87	0.96	0 ± 1.84	< 0.001
HR	0.88	0.77	1.26	0 ± 2.44	< 0.001
Acti-Heart	0.81	0.65	1.53	0.21 ± 4.28	< 0.001
**Control Group**					
GT3X+ and HR	0.91	0.83	0.48	0 ± 0.93	< 0.001
HR	0.84	0.71	0.62	0 ± 1.82	< 0.001
Acti-Heart	0.67	0.45	0.85	0.29 ± 1.82	< 0.001

Across all treadmill walking tasks, the HR response in individuals with unilateral amputation was 1.3 to 1.5 times greater than the physically active control group. At treadmill speeds between 1 to 2mph and a gradient of 3% at 2 mph, the HR response of individuals with bilateral amputation was 1.5 to 1.7 times greater than control participants. At these same walking speeds the HR of the bilateral amputation group was between 1.1 to 1.3 times greater than the group with unilateral amputation. These differences in HR increase linearly with increases in treadmill intensity (Table 2). The mean PCI across all velocities for each group ranged from 0.67 to 1.01, 1.03 to 1.45 and 0.38 to 0.61 in the unilateral, bilateral and control group, respectively.

The population specific equations (GT3X+HR) to predict ambulatory PAEE are described below:

Unilateral amputation: *PAEE = (0*.000453*PAC) + (0.045487*HR) - 2.*713284*

Bilateral amputation: *PAEE = (0*.000658*PAC) + (0.025308*HR) - 1.*795157*

Controls: *PAEE = (0*.000550*PAC) + (0.036472*HR) - 1.*797866*

**Fig 1:**
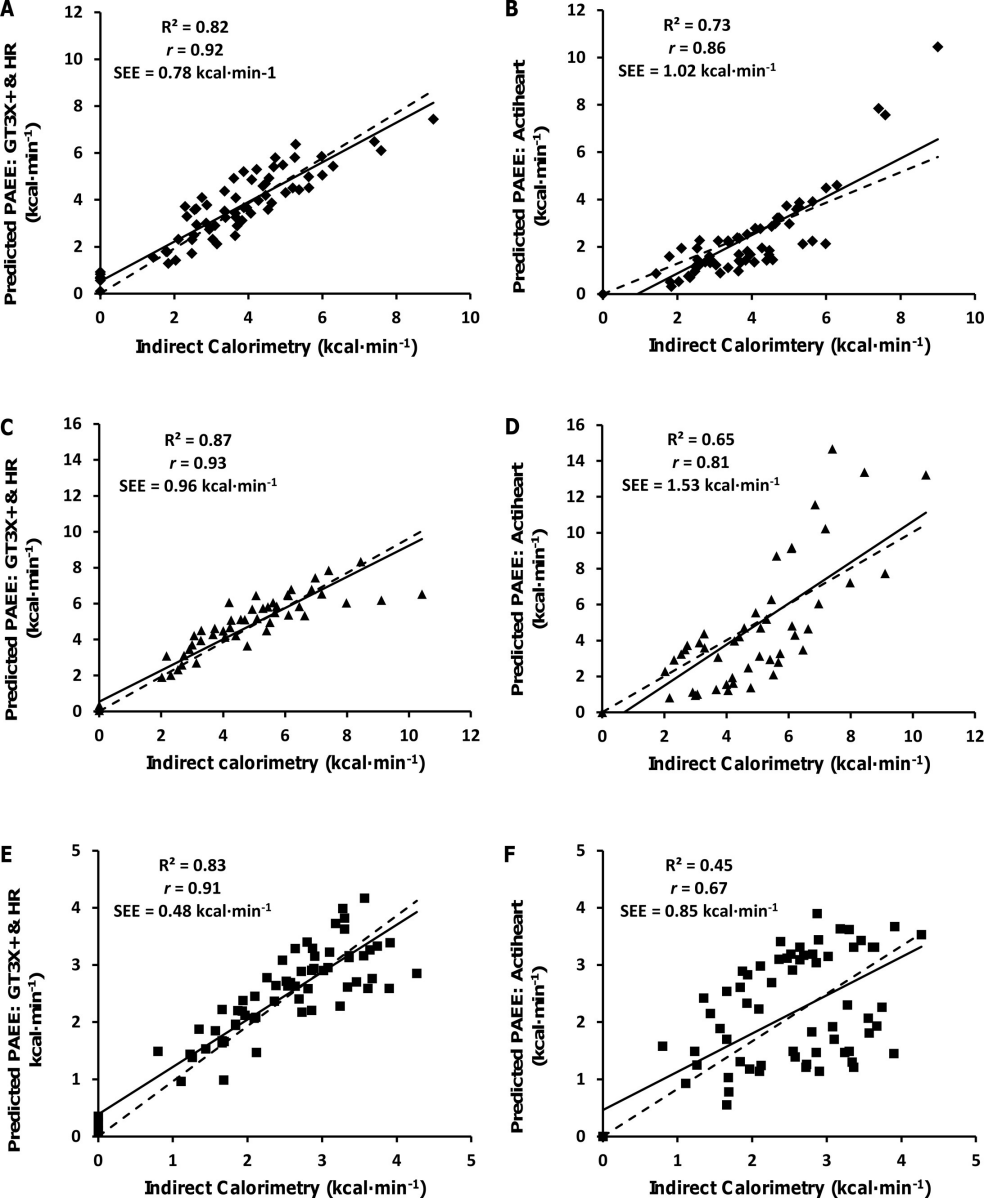
Scatterplots showing the relationship between estimated and criterion PAEE. Estimated PAEE from GT3X+ with heart rate and criterion PAEE (A, C, E). Estimated PAEE from the Actiheart and criterion PAEE (B, D, F). The scatterplots show the unilateral amputation group (A, B), bilateral amputation group (C, D) and the non-injured control group (E, F). The straight line represents the models best fit, and the dotted line indicates the line of identity.

**Fig 2:**
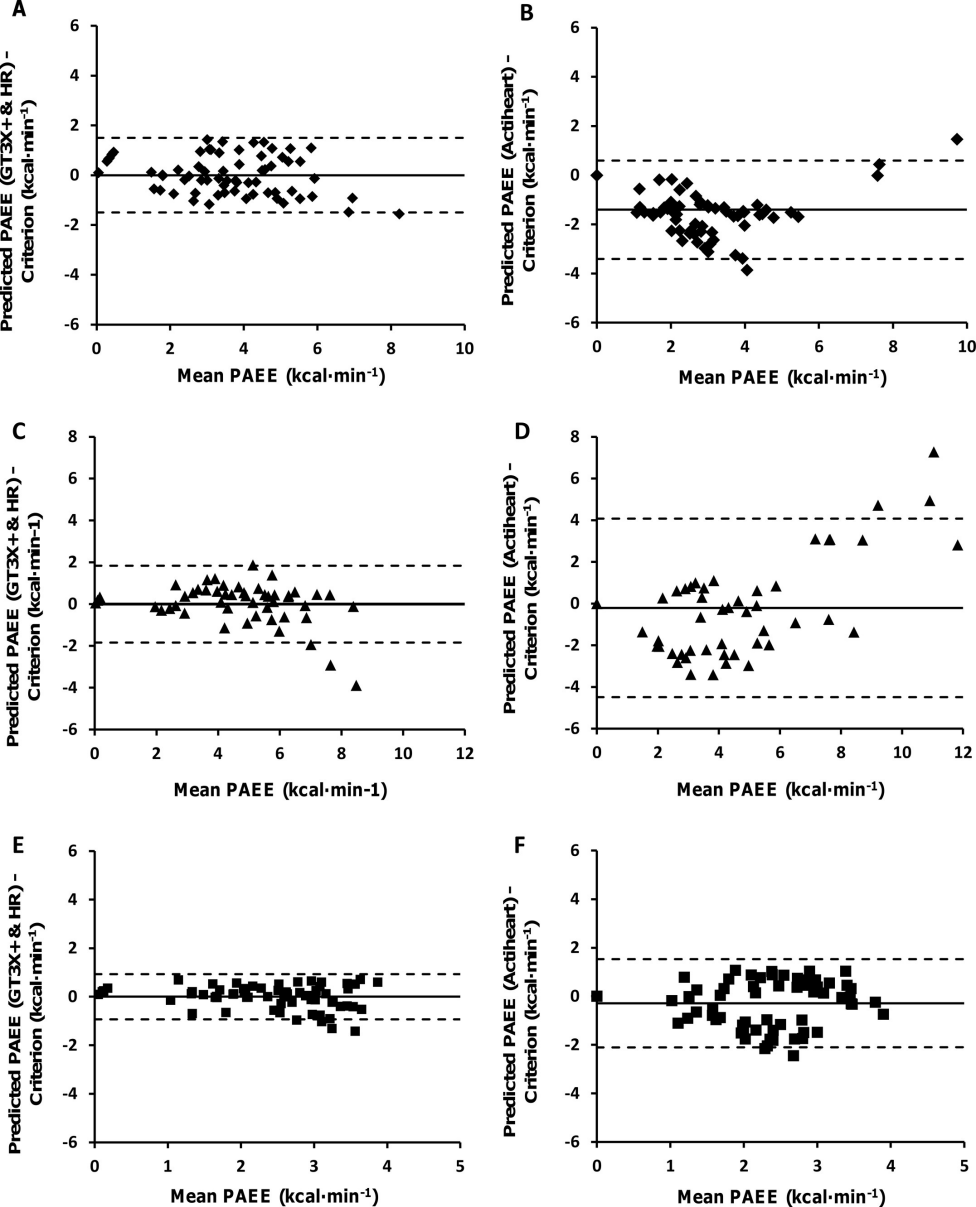
**Bland and Altman plots for the criterion and predicted PAEE using the GT3X+ with heart rate estimation models (A, C, E), and criterion and predicted PAEE using the Actiheart (B, D, F).** The plots show the unilateral group (A, B), bilateral group (C, D) and non-injured control group (E, F). The straight line demonstrates the mean and the dotted line indicates the 95% Limits of Agreement (LoA).

The relationships between criterion PAEE and predicted PAEE, derived from GT3X+HR and AHR, are presented as scatter plots in Fig 1. Fig 2 illustrates the mean bias and 95% limits of agreement (LoA) differences, when comparing the criterion PAEE data with estimated PAEE derived from population specific prediction models (GT3X+HR) and the proprietary group calibration algorithm of the AHR. When comparing both methods across all groups the GT3X+HR demonstrates the smallest LoA and the AHR shows the greatest LoA. When comparing populations, the control group demonstrate the smallest LoA and the bilateral group demonstrate the largest LOA for both GT3X+HR and AHR methods (Table 3).

**Fig 3:**
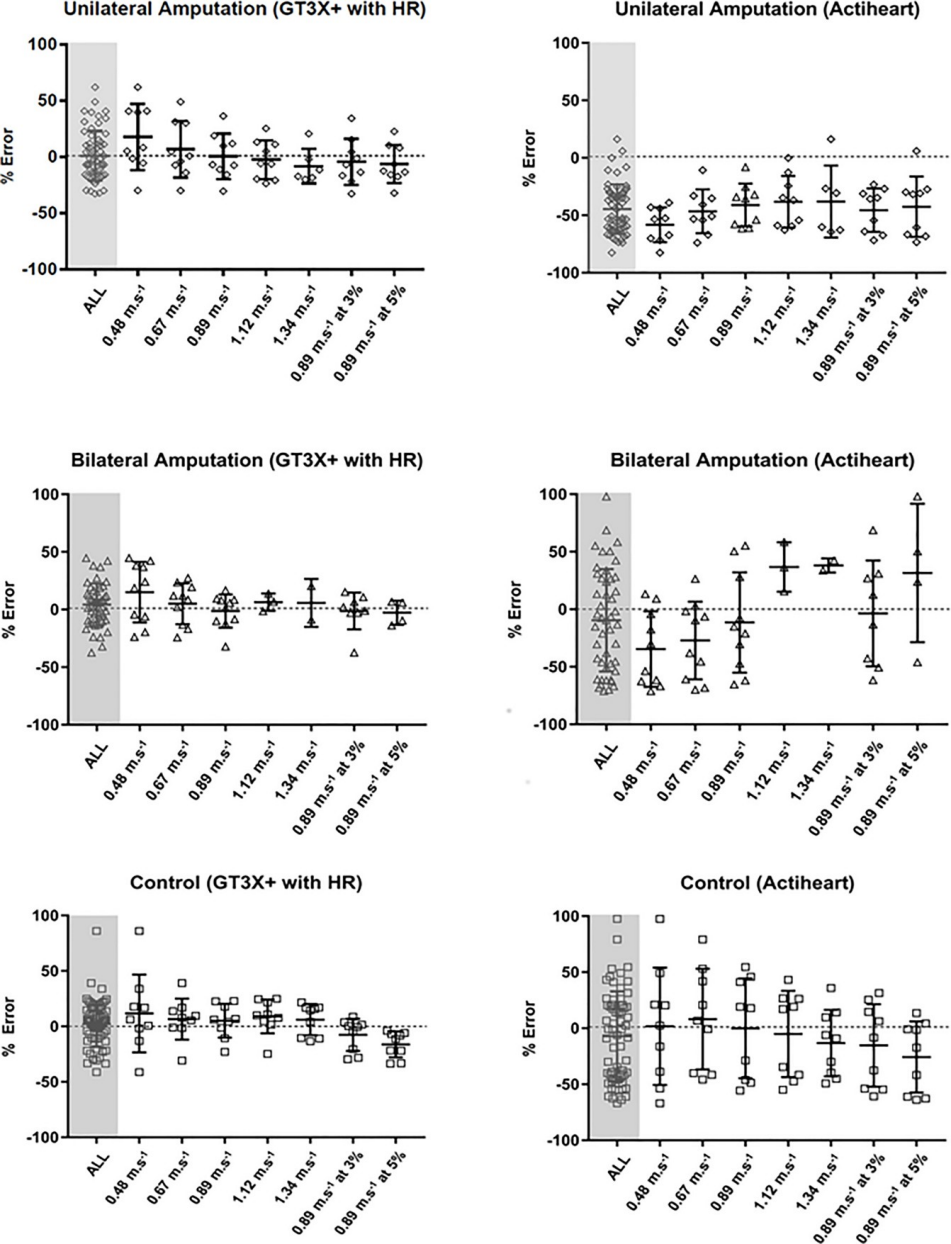
**Modified box and whisker plots demonstrating the mean percentage error of estimation relative to criterion for each treadmill activity using GT3X+ with heart rate estimation models (A, C, E), and Actiheart (B, D, F).** The plots show the unilateral group (A, B), bilateral group (C, D) and control group (E, F).

Fig 3 demonstrates modified box and whisker plots depicting the mean percentage error of estimation relative to criterion for each treadmill activity using the cross validated, GT3X+HR model against the pre-determined algorithm used in the AHR device. Error statistics between the criterion and predicted PAEE from the GT3X+HR model, HR alone and AHR for each treadmill task are shown in S1 Table.

Participants were asked to complete 35 minutes of walking in total. Participants reasoning for discontinuing with the trial were not asked. However, a large proportion of participants stated an uncomfortable stump due to repeated impact and friction inside their socket. For individuals with transfemoral bilateral amputation the inability to walk quickly enough in their prosthesis without the risk of falling was also reported. Discontinuing with the trial due to excessive physical exertion or fatigue was less common, as demonstrated by the RPE scores in Table 2. The RER values during the walking tasked range from a mean 0.76 ± 0.04 to 0.86 ± 0.05 and 0.78 ± 0.04 to 0.93 ± 0.02 in the unilateral and bilateral amputation groups respectively. RER values ranged from 0.78 ± 0.03 to 0.84 ± 0.04 in the control group.

## Discussion

This study is a controlled laboratory validation study to assess the accuracy of a research grade multi-sensor device (AHR) and a population specific manually derived equation, which incorporated both HR and PAC (GT3X+HR), to predict ambulatory PAEE in individuals lower-limb amputation/s. Furthermore, we were able to assess the validity of these methods on both unilateral and bilateral lower-limb amputation groups, to assess the generalizability of these methods across this specific population. Of the two multi-sensor activity methods considered in this study, these data indicate the GT3X+HR model, integrating accelerometry data from an Actigraph GT3X+ worn at the hip of the shortest residual limb, with HR data, provided the most valid estimation of ambulatory PAEE in both amputation groups. HR signals alone produced superior accuracy for the estimation of PAEE than the AHR device. Therefore, the proprietary group calibration algorithm, intrinsic to the AHR, has lower validity when predicting PAEE in individuals with unilateral or bilateral transtibial and transfemoral amputations, potentially because the algorithms are not optimized for this population. The results of this study also demonstrate that level of amputation impacts on the ability to accurately predict PAEE, with all multi-sensor methods displaying greater LoA and SEE values in unilateral versus control and in bilateral versus unilateral (Table 3).

HR has benefits as a physiological variable as it increases linearly and proportionately with exercise intensity and thus metabolic rate [32]. HR alone in this study explains 79%, 77% and 71% of the variance in the unilateral, bilateral and control group respectively. The reason HR may have performed so well is because it is highly individualised to the groups of interest and the task intensities were linear in nature. As HR at lower exercise intensities is affected by other factors, such as psychological or thermal stress, it would be intuitive to hypothesise that the integration of acceleration values may offer a more reliable estimation of PAEE. The findings of this current study demonstrate evidence of this when combined with the PAC from the GT3X+. However, the AHR, a multi-sensor device which uses proprietary group calibration algorithms intrinsic to the device, explains less of the variance and demonstrates greater error in the estimation of ambulatory PAEE than HR alone. The proprietary equations, derived from Brage *et al*. (2004) [31] and utilised here were designed to predict EE during ambulation in able-bodied healthy adults, not those with lower-limb impairments or significant gait abnormalities. The AHR uses a uni-axial accelerometer unlike the GT3X+ which is a tri-axial accelerometer, uni-axial accelerometers have been shown to have less sensitivity when predicting PAEE [39]. It is also known that the anatomical location of an accelerometer is important in the accuracy of measuring PAEE [19, 40]. The anatomical locations of the monitors used in this study are not comparable, as the AHR device was worn on the chest whilst the GT3X+ was worn on the hip of the shortest residual limb. It may be that there is reduced sensitivity with accelerometers being worn at the chest compared to hip during ambulatory movements. The activity protocol adopted in this study captured a wide range of walking speeds, which are representative of the exercise intensities for lower-limb amputation groups undergoing in-patient rehabilitation. The relative exercise intensities were consequently much lower for healthy able-bodied controls (Table 2). The weaker correlations in the control group compared to the amputation groups may be an artefact of there being a narrower range of exercise intensities. Although not necessarily an accurate reflection of the general population, they provide normative values for physically active military personnel, thereby allowing us to draw closer comparisons to the groups of individuals with an amputation(s) pre-injury and peer-group PAEE values.

Our previous research identified that wearing a GT3X+ on the hip of the shortest residual limb as the most accurate anatomical position around the pelvis to predict PAEE in individuals with unilateral and bilateral amputation [19]. When activity counts from the GT3X+ were combined with significant covariates this created population specific estimation models, capable of accurately estimating ambulatory PAEE (mean absolute percentage error); unilateral (21±17%), bilateral (16±15%) and controls (15±7%). When compared to our current findings (Table 3), the incorporation of a physiological variable (HR) with the GT3X+ monitor in the unilateral group created stronger correlations, explained greater amounts of variance and displayed lower random error than the GT3X+ with covariates (level of amputation and length of rehabilitation). In the bilateral amputation and control groups the GT3X+HR model and the GT3X+ with covariates models (bilateral amputation group covariates; waist circumference, and control; body mass) were very similar.

Previous research has focused on determining the effects of different prosthetic components and design on the economy of gait in individuals with amputation [18]. Comparing gait, prosthetic fitting and components in a controlled laboratory environment is an important field of study in the development of ambulatory function in individuals with amputation. How these prosthetics function outside of the laboratory however is of great importance to researchers, manufacturers, clinicians and patients. Research in the area of assessing mobility in individuals with amputation has relied on subjective self-reported questionnaires [14, 15]. However, subjective measurements of PA, although low cost, applicable to large populations and practical [41], are commonly found to demonstrate low-moderate predictive validity [42] and lack sensitivity [41] when compared to objective measures of PA. The objective model of estimating PAEE that we have developed for this study and previously [19] could provide insight into the amount of habitual ambulation-related PAEE individuals with amputations engage in, whilst wearing a range of prosthetic devices outside of the laboratory.

Unfavourable body composition changes can occur after amputation [4–6] and individuals with amputation are at increased risk of developing cardio-metabolic disorders [1–3] and a number of other secondary physical conditions [43], including osteoporosis, low back pain [44] and osteoarthritis [45]. The models we have developed could allow clinicians and researchers to objectively assess the influence of different environments (e.g. weather and terrain or urban versus rural living conditions) and rehabilitation settings (e.g. in-patient versus home-based) on PAEE. Future investigations may provide insight into different vocational prospects in the community, thus optimising the potential for full integration back into society.

As wearable consumer PA monitors devices become more commonplace there are greater opportunities for people to engage in the self-management of their own care as well as providing lifestyle information to health care providers [46]. As a note of comparison, the absolute percentage error of consumer multisensory devices (e.g. Fitbit, Microsoft Band and Apple Watch) [24] ranged from 24 to 73% during walking tasks in healthy adults. In this study using individuals with amputation, the accuracy of the research grade AHR demonstrated a comparable level of accuracy (20–28%). The GT3X+HR models developed in individuals with lower-limb amputation demonstrate a superior level of accuracy (12–14%). Despite the welcomed potential utility of these commercially available multi-sensor devices, until they have been validated in people with lower limb-loss it would be ill-advised to recommend there use for the accurate measurement and monitoring of PA in these groups. While our PAEE estimation models show promising levels of accuracy, the wider scientific community needs to agree upon a threshold to signify an acceptable level of accuracy when using wearable devices.

A limitation of this study is the relatively small sample size and variations within participants based on the diversity in the severity of lower-limb loss injuries. However, this diversity may be considered beneficial as the range of walking abilities captured improves the external validity of the regression equations, making them more suitable for the wider amputee population. Also, despite the diversity of the population, the amount of unexplained random error is relatively small. The inclusion of a diverse range of participants is in accordance with best practice recommendations for PA validation studies [47]. Although the participants in this study are military personnel, the injuries sustained in our unilateral group are not dissimilar to what might be expected in a road traffic accident or adventure sports. We therefore believe that our findings are applicable to physically active civilian cohorts with lower-limb amputation population. It is important to note that participants were not provided with a familiarisation of treadmill walking prior to starting the trial. However, many would have been exposed to treadmill walking/running as part of their rehabilitation or as their preferred mode of cardiorespiratory exercise whilst at home. We acknowledge that for some individuals (primarily the bilateral ampution group) this may have affected the energy expenditure data due to the lack of familiarity with the exercise task. However, it is likely that the GT3X+ still managed to assess increased movement due to atypical gait, thus increased corresponding GT3X+ and HR outputs. This study did not measure self-selected walking speed over-ground. There may have been differences in the energy cost of walking over-ground compared with treadmill [48], therefore potentially reducing the accuracy of using these generated equations in the assessment of free-living ambulation. It is perhaps unsurprising that a bespoke algorithm, developed for this population specifically, performed better than an algorithm that was generated on a completed independent and physiologically different sample. Nevertheless, if the Actiheart device is to be applicable to various clinical populations with atypical gait patterns and asymmetries, alternative proprietary equations and/or independent HR calibrations appear necessary [49].

The measurement of PAEE has proven inherently difficult to measure, even in humans without mobility-related physical impairments. Commercial and research grade PA sensors/algorithms are unlikely to have been developed with the movement characteristics and energy demands of amputee populations in mind. We feel population specific algorithms are important to account for the numerous mobility or physical barriers individuals with amputation are likely to encounter when engaging in physical activity. An individualised approach would improve accuracy, either from a cut-point [50] or HR and PAEE relationship perspective. This approach is more time consuming and not necessarily feasible for large scale trials.

Future research should consider applying and further developing new data analysis techniques such as artificial neural networks [51, 52], hidden Markov models [53] and classification trees [54] which use the rich information to classify certain activities and derive a more accurate estimate of EE. Future models should consider using additional activities (i.e. not just walking) that better resemble free-living conditions for individuals with amputation and evaluating the performance of EE prediction models during recovery after exercise (which contributes to TDEE). We encourage research groups to work collaboratively to recruit larger sample sizes. Using a large diverse samples of participants and with different aetiologies would provide a more robust model for the assessment of PAEE in individuals with amputations. Future studies should also aim to cross-validate these newly developed population specific equations using a completely independent sample of participants, across a range of activities of daily living or during habitual free-living conditions.

## Conclusion

The manually derived model, integrating HR and acceleration data, from a device positioned at the hip of the shortest residual limb (Actgraph GT3X+), has been shown to possess greater validity for estimating ambulatory-related PAEE in persons with traumatic lower-limb amputations, compared to the proprietary group calibrated algorithm intrinsic to the AHR device. Due to the poorer predictive validity of the AHR device, we would recommend using the GT3X+ HR, together with the algorithms presented in this article, which demonstrate lower unexplained variance and lower estimation error.

## Supporting information

S1 Table Mean absolute error ((MAE); kcal·min^-1^), mean absolute percentage error and root mean squared error (RMSE) of predicted PAEE using the GT3X+ with HR, HR signals alone and Actiheart. Data expressed as mean ± SD.
(DOCX)

S2 Table Results from the one-way ANOVA demonstrating between group differences during each activity for criterion PAEE (Metamax 3b), Actigraph GT3X+, heart rate, Actiheart, METs and physiological cost index (PCI). Data expressed as mean ± SD.
(DOCX)
