# Supplementary material for: Predicting ambulatory energy expenditure in lower limb amputees using multi-sensor methods
Source: PLoS One. 2019 Jan 31;14(1):e0209249. doi: 10.1371/journal.pone.0209249 (PMC6354995; doi:10.1371/journal.pone.0209249)
Supplement: S1 Table — Data expressed as mean ± SD. (DOCX) [file pone.0209249.s001.docx]

Supplementary Table 1: Mean absolute error (MAE); kcal·min^-1^), mean absolute percentage error and root mean squared error (RMSE) of predicted PAEE using the generated models of the GT3X+ with HR, HR signals alone and Actiheart^TM^. Data expressed as mean ± SD

| **Activity** | **MAE (kcal·min^-1^)** | | | | | | | | |
| --- | --- | --- | --- | --- | --- | --- | --- | --- | --- |
|  | **Unilateral Amputees** | | | **Bilateral Amputees** | | | **Control** | | |
|  | **GT3X+ and HR** | **HR** | **Acti-Heart** | **GT3X+ and HR** | **HR** | **Acti-Heart** | **GT3X+ and HR** | **HR** | **Acti-Heart** |
| **Resting** | 0.53 ± 0.28 | 1.00 ± 0.73 | 0.00 ± 0.00 | 0.22 ± 0.16 | 0.70 ± 0.50 | 0.00 ± 0.00 | 0.20 ± 0.11 | 0.35 ± 0.24 | 0.00 ± 0.00 |
| **0.48 m.s^-1^** | 0.61 ± 0.51 | 0.78 ± 0.49 | 1.37 ± 0.41 | 0.94 ± 0.53 | 1.06 ± 0.75 | 1.42 ± 1.06 | 0.30 ± 0.28 | 0.51 ± 0.50 | 0.55 ± 0.36 |
| **0.67 m.s^-1^** | 0.56 ± 0.47 | 0.59 ± 0.46 | 1.40 ± 0.59 | 0.76 ± 0.55 | 0.93 ± 0.83 | 1.36 ± 1.08 | 0.25 ± 0.22 | 0.41 ± 0.36 | 0.66 ± 0.38 |
| **0.89 m.s^-1^** | 0.59 ± 0.38 | 0.47 ± 0.46 | 1.46 ± 0.62 | 0.69 ± 0.81 | 1.08 ± 1.06 | 1.99 ± 1.04 | 0.30 ± 0.21 | 0.43 ± 0.30 | 0.92 ± 0.39 |
| **1.12 m.s^-1^** | 0.65 ± 0.44 | 0.64 ± 0.50 | 1.55 ± 0.93 | 0.29 ± 0.15 | 0.79 ± 0.30 | 2.26 ± 2.32 | 0.41 ± 0.29 | 0.40 ± 0.45 | 0.99 ± 0.49 |
| **1.34 m.s^-1^** | 0.87 ± 0.49 | 1.11 ± 0.54 | 2.37 ± 1.04 | 0.66 ± 0.02 | 0.69 ± 0.19 | 2.08 ± 1.37 | 0.47 ± 0.16 | 0.57 ± 0.47 | 0.89 ± 0.58 |
| **3% gradient**  **at 0.89 m.s^-1^** | 0.67 ± 0.42 | 0.58 ± 0.55 | 1.85 ± 0.70 | 0.75 ± 1.29 | 1.06 ± 1.25 | 2.23 ± 1.38 | 0.34 ± 0.41 | 0.51 ± 0.37 | 0.94 ± 0.62 |
| **5% gradient**  **At 0.89 m.s^-1^** | 0.73 ± 0.32 | 0.69 ± 0.47 | 1.97 ± 1.01 | 0.47 ± 0.10 | 0.92 ± 0.39 | 3.51 ± 2.72 | 0.60 ± 0.49 | 0.55 ± 0.50 | 1.05 ± 0.99 |
| **All Activities** | **0.64 ± 0.41** | **0.72 ± 0.54** | **1.46 ± 0.94** | **0.64 ± 0.69** | **0.94 ± 0.81** | **1.59 ± 1.50** | **0.36 ± 0.31** | **0.47 ± 0.40** | **0.75 ± 0.61** |
| **Activity** | **Mean absolute percentage error (%)** | | | | | | | | |
|  | **Unilateral Amputees** | | | **Bilateral Amputees** | | | **Control** | | |
|  | **GT3X+ and HR** | **HR** | **Acti-Heart** | **GT3X+ and HR** | **HR** | **Acti-Heart** | **GT3X+ and HR** | **HR** | **Acti-Heart** |
| **Resting** | - | - | - | - | - | - | - | - | - |
| **0.48 m.s^-1^** | 26 ± 21.6 | 35 ± 21.5 | 58 ± 15.2 | 26 ± 14.3 | 27 ± 13.7 | 39 ± 26.7 | 24 ± 27.0 | 42 ± 54.6 | 41 ± 29.6 |
| **0.67 m.s^-1^** | 19 ± 16.6 | 21 ± 18.1 | 47 ± 19.1 | 16 ± 7.9 | 19 ± 11.9 | 33 ± 27.4 | 14 ± 13.1 | 23 ± 24.7 | 37 ± 24.1 |
| **0.89 m.s^-1^** | 17 ± 10.4 | 15 ± 15.6 | 41 ± 18.6 | 11 ± 8.3 | 18 ± 11.8 | 38 ± 20.4 | 13 ± 8.8 | 19 ± 14.9 | 40 ± 14.4 |
| **1.12 m.s^-1^** | 15 ± 8.0 | 16 ± 14.4 | 38 ± 22.5 | 7 ± 6.2 | 15 ± 3.6 | 37 ± 21.5 | 15 ± 9.4 | 14 ± 13.7 | 35 ± 13.1 |
| **1.34 m.s^-1^** | 15 ± 7.1 | 21 ± 14.4 | 43 ± 21.3 | 15 ± 8.1 | 14 ± 3.8 | 38 ± 6.1 | 14 ± 5.0 | 16 ± 12.1 | 27 ± 16.9 |
| **3% gradient at 0.89 m.s^-1^** | 16 ± 11.7 | 16 ± 15.75 | 46 ± 18.9 | 10 ± 12.1 | 16 ± 11.2 | 38 ± 21.1 | 11 ± 12.0 | 17 ± 10.4 | 33 ± 20.6 |
| **5% gradient at 0.89 m.s^-1^** | 15 ± 7.9 | 14 ± 12.1 | 44 ± 23.7 | 9 ± 3.5 | 16 ± 4.3 | 55 ± 31.2 | 16 ± 11.7 | 15 ± 11.6 | 30 ± 27.6 |
| **All Activities** | **18 ± 13.9** | **20 ± 16.9** | **45 ± 19.9** | **15 ± 11.5** | **19 ± 11.5** | **39 ± 23.4** | **15 ± 13.9** | **21 ± 25.5** | **34 ± 27.6** |
| **Activity** | **Root Mean Square Error (RMSE) (kcal·min^-1^)** | | | | | | | | |
|  | **Unilateral Amputees** | | | **Bilateral Amputees** | | | **Control** | | |
|  | **GT3X+ and HR** | **HR** | **Acti-Heart** | **GT3X+ and HR** | **HR** | **Acti-Heart** | **GT3X+ and HR** | **HR** | **Acti-Heart** |
| **Resting** | 0.59 | 1.21 | 0.00 | 0.27 | 0.85 | 0.00 | 0.22 | 0.42 | 0.00 |
| **0.48 m.s^-1^** | 0.78 | 0.91 | 1.43 | 1.06 | 1.28 | 1.74 | 0.40 | 0.70 | 0.65 |
| **0.67 m.s^-1^** | 0.72 | 0.73 | 1.50 | 0.92 | 1.22 | 1.70 | 0.32 | 0.53 | 0.75 |
| **0.89 m.s^-1^** | 0.69 | 0.64 | 1.58 | 1.03 | 1.47 | 2.22 | 0.36 | 0.52 | 0.99 |
| **1.12 m.s^-1^** | 0.77 | 0.80 | 1.78 | 0.31 | 0.82 | 2.95 | 0.50 | 0.58 | 1.10 |
| **1.34 m.s^-1^** | 0.98 | 1.22 | 2.56 | 0.66 | 0.70 | 2.29 | 0.50 | 0.72 | 1.04 |
| **3% gradient at 0.89 m.s^-1^** | 0.78 | 0.78 | 1.97 | 1.42 | 1.58 | 2.57 | 0.51 | 0.62 | 1.11 |
| **5% gradient at 0.89 m.s^-1^** | 0.79 | 0.81 | 2.19 | 0.48 | 0.98 | 4.23 | 0.76 | 0.72 | 1.40 |
| **All Activities** | **0.76** | **0.90** | **1.73** | **0.93** | **1.24** | **2.18** | **0.47** | **0.61** | **0.96** |
