# Supplementary material for: Predicting ambulatory energy expenditure in lower limb amputees using multi-sensor methods
Source: PLoS One. 2019 Jan 31;14(1):e0209249. doi: 10.1371/journal.pone.0209249 (PMC6354995; doi:10.1371/journal.pone.0209249)
Supplement: S2 Table — Data expressed as mean ± SD. (DOCX) [file pone.0209249.s002.docx]

| **Activity** | **Between Group One-Way ANOVA** |
| --- | --- |
| **Resting Metabolic Rate (kcal·min^-1^)** | F(2, 25) = 3.46, p =0.047 |
| **PAEE Metamax 3b (kcal·min^-1^) at 0.48 m.s^-1^** | F(2, 25) = 14.50, p < 0.0001 |
| **PAEE Metamax 3b (kcal·min^-1^) at 0.67 m.s^-1^** | F(2, 25) = 15.74, p < 0.0001 |
| **PAEE Metamax 3b (kcal·min^-1^) at 0.89 m.s^-1^** | F(2, 25) = 15.48, p < 0.0001 |
| **PAEE Metamax 3b (kcal·min^-1^) at 1.12 m.s^-1^** | F(2, 18) = 5.59, p = 0.013 |
| **PAEE Metamax 3b (kcal·min^-1^) at 1.34 m.s^-1^** | F(2, 14) = 7.20, p = 0.007 |
| **PAEE Metamax 3b (kcal·min^-1^) 3% gradient at 0.89 m.s^-1^** | F(2, 23) = 9.65, p = 0.001 |
| **PAEE Metamax 3b (kcal·min^-1^) 5% gradient at 0.89 m.s^-1^** | F(2, 19) = 6.76, p = 0.006 |
| **ActiGraph GT3X+ (PAC·min^-1^) at 0.48 m.s^-1^** | F(2, 25) = 29.50, p < 0.0001 |
| **ActiGraph GT3X+ (PAC·min^-1^) at 0.67 m.s^-1^** | F(2, 25) = 24.10, p < 0.0001 |
| **ActiGraph GT3X+ (PAC·min^-1^) at 0.89 m.s^-1^** | F(2, 25) = 22.48, p < 0.0001 |
| **ActiGraph GT3X+ (PAC·min^-1^) at 1.12 m.s^-1^** | F(2, 18) = 7.293, p = 0.005 |
| **ActiGraph GT3X+ (PAC·min^-1^) at 1.34 m.s^-1^** | F(2, 14) = 5.88, p = 0.014 |
| **ActiGraph GT3X+ (PAC·min^-1^) 3% gradient at 0.89 m.s^-1^** | F(2, 23) = 22.11, p < 0.0001 |
| **ActiGraph GT3X+ (PAC·min^-1^) 5% gradient at 0.89 m.s^-1^** | F(2, 19) = 10.79, p = 0.001 |
| **Heart rate (beats·min^-1^) at 0.48 m.s^-1^** | F(2, 25) = 18.96, p < 0.0001 |
| **Heart rate (beats·min^-1^) at 0.67 m.s^-1^** | F(2, 25) = 27.70, p < 0.0001 |
| **Heart rate (beats·min^-1^) at 0.89 m.s^-1^** | F(2, 25) = 26.29, p < 0.0001 |
| **Heart rate (beats·min^-1^) at 1.12 m.s^-1^** | F(2, 18) = 11.68, p = 0.001 |
| **Heart rate (beats·min^-1^) at 1.34 m.s^-1^** | F(2, 14) = 9.46, p = 0.003 |
| **Heart rate (beats·min^-1^) 3% gradient at 0.89 m.s^-1^** | F(2, 23) = 23.72, p < 0.0001 |
| **Heart rate (beats·min^-1^) 5% gradient at 0.89 m.s^-1^** | F(2, 19) = 14.87, p < 0.0001 |
| **ActiHeart (kcal·min^-1^) at 0.48 m.s^-1^** | F(2, 25) = 5.69, p = 0.009 |
| **ActiHeart (kcal·min^-1^) at 0.67 m.s^-1^** | F(2, 25) = 5.20, p = 0.013 |
| **ActiHeart (kcal·min^-1^) at 0.89 m.s^-1^** | F(2, 25) = 6.74, p = 0.005 |
| **ActiHeart (kcal·min^-1^) at 1.12 m.s^-1^** | F(2, 18) = 7.07, p = 0.005 |
| **ActiHeart (kcal·min^-1^) at 1.34 m.s^-1^** | F(2, 14) = 2.86, p = 0.091 |
| **ActiHeart (kcal·min^-1^) 3% gradient at 0.89 m.s^-1^** | F(2, 23) = 5.73, p = 0.010 |
| **ActiHeart (kcal·min^-1^) 5% gradient at 0.89 m.s^-1^** | F(2, 19) = 6.39, p = 0.008 |
| **MET at 0.48 m.s^-1^** | F(2, 25) = 15.45, p < 0.0001 |
| **MET at 0.67 m.s^-1^** | F(2, 25) = 16.25, p < 0.0001 |
| **MET at 0.89 m.s^-1^** | F(2, 25) = 15.80, p < 0.0001 |
| **MET at 1.12 m.s^-1^** | F(2, 18) = 6.20, p = 0.009 |
| **MET at 1.34 m.s^-1^** | F(2, 14) = 6.07, p = 0.013 |
| **MET 3% gradient at 0.89 m.s^-1^** | F(2, 23) = 10.08, p = 0.001 |
| **MET 5% gradient at 0.89 m.s^-1^** | F(2, 19) = 7.22, p = 0.005 |
| **Physiological Cost Index at 0.48 m.s^-1^** | F(2, 25) = 23.97, p < 0.0001 |
| **Physiological Cost Index at 0.67 m.s^-1^** | F(2, 25) = 34.18, p < 0.0001 |
| **Physiological Cost Index at 0.89 m.s^-1^** | F(2, 25) = 27.24, p < 0.0001 |
| **Physiological Cost Index at 1.12 m.s^-1^** | F(2, 18) = 14.70, p < 0.0001 |
| **Physiological Cost Index at 1.34 m.s^-1^** | F(2, 14) = 8.89, p = 0.003 |
| **Physiological Cost Index: 3% gradient at 0.89 m.s^-1^** | F(2, 25) = 19.49, p < 0.0001 |
| **Physiological Cost Index: 5% gradient at 0.89 m.s^-1^** | F(2, 19) = 9.86, p = 0.001 |

A significant difference in criterion PAEE, HR and METs were only reported at higher intensities (1.12 m.s^-1^, 1.34 m.s^-1^ and 5% gradient at 0.89 m.s^-1^) between individuals with unilateral amputation and control group (*P*<0.05).

A significant differences in criterion PAEE, HR, METs, PAC (GT3X+ worn at the longest and shortest limb) and PCI were found between bilateral amputation versus the unilateral and control groups at all speeds analysed (*P*<0.05).

Significant differences in PAC (GT3X+) were only reported at the lowest intensity of **0.48 m.s^-1^** and the highest intensity of **1.34 m.s^-1^** between individuals with unilateral amputation and control group (P<0.05).

Significant differences in Actiheart outcomes were reported between the bilateral amputation and the unilateral amputation groups at all speeds analysed and at 0.89 m.s^-1^ and 3% gradient at 0.89 m.s^-1^ versus the control group.

Significant differences in PCI were only reported at the lowest intensities (0.48 m.s^-1^ and 0.67 m.s^-1^) and highest intensities (1.34 m.s^-1^ and 0.89 m.s^-1^) between individuals with unilateral amputation and the control group
